# Metal Organic Frameworks as Desulfurization Adsorbents of DBT and 4,6-DMDBT from Fuels

**DOI:** 10.3390/molecules24244525

**Published:** 2019-12-10

**Authors:** Zoi-Christina Kampouraki, Dimitrios A. Giannakoudakis, Vaishakh Nair, Ahmad Hosseini-Bandegharaei, Juan Carlos Colmenares, Eleni A. Deliyanni

**Affiliations:** 1 Laboratory of Chemical and Environmental Technology, Chemistry Department, Aristotle University of Thessaloniki, GR–541 24 Thessaloniki, Greece; zoiikamp@gmail.com; 2 Institute of Physical Chemistry, Polish Academy of Sciences, Kasprzaka 44/52, 01-224 Warsaw, Poland; jcarloscolmenares@ichf.edu.pl; 3 Department of Chemical Engineering, National Institute of Technology Karnataka (NITK), Surathkal, Srinivasanagar P.O. Mangalore 575025, India; vaishakhchem@gmail.com; 4 Department of Environmental Health Engineering, Faculty of Health, Sabzevar University of Medical Sciences, Sabzevar POB 319, Iran; ahoseinib@yahoo.com; 5 Department of Engineering, Kashmar Branch, Islamic Azad University, PO Box 161, Kashmar, Iran

**Keywords:** metal organic framework (MOF), adsorptive desulfurization of fuels, thiophenic compounds, dibenzothiophene (DBT), 4,6-dimethyldibenzothiophene (4,6-DMDBT)

## Abstract

Ultradeep desulfurization of fuels is a method of enormous demand due to the generation of harmful compounds during the burning of sulfur-containing fuels, which are a major source of environmental pollution. Among the various desulfurization methods in application, adsorptive desulfurization (ADS) has low energy demand and is feasible to be employed at ambient conditions without the addition of chemicals. The most crucial factor for ADS application is the selection of the adsorbent, and, currently, a new family of porous materials, metal organic frameworks (MOFs), has proved to be very effective towards this direction. In the current review, applications of MOFs and their functionalized composites for ADS are presented and discussed, as well as the main desulfurization mechanisms reported for the removal of thiophenic compounds by various frameworks. Prospective methods regarding the further improvement of MOF’s desulfurization capability are also suggested.

## 1. Introduction

**Figure 1:**
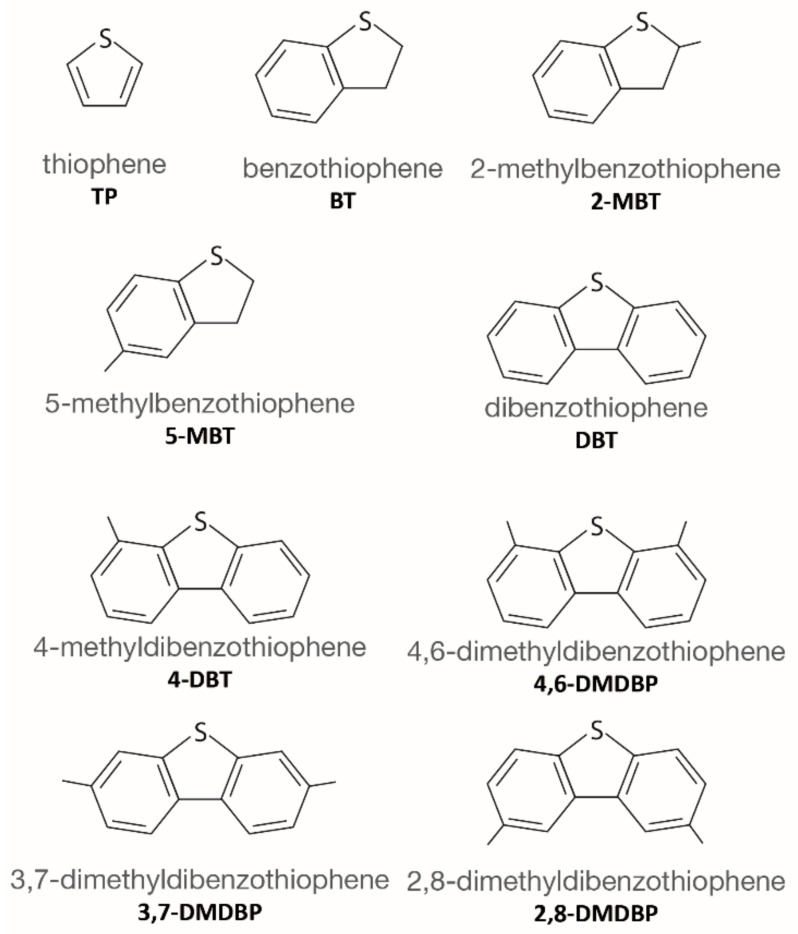
The most important thiophene derivatives.

Fossil fuels are the most commonly used source of energy all around the world; however, the emission of hazardous and dangerous chemical substances during their use is an important threat to the human society as well as the environment [1]. Crude oil, gasoline, diesel, jet fuel, and furnace oil are some of the fossil-derived fuels which contain nitrogen and sulfur compounds (NCs and SCs, respectively), which, during combustion, produce hazardous oxides such as SO_x_, NO_x_, and CO_2_. The major SCs found in these fuels, collected in Figure 1, are thiophene (TP) and its derivatives like benzothiophene (BT), 2-methylbenzothiophene (2-MBT), 5-methylbenzothiophene (5-MBT), dibenzothiophene (DBT), 4,6-dimethyldibenzothiophene (4,6-DMDBT), 3,7-dimethyldibenzothiophene (3,7-DMDBT), and 2,8-dimethyldibenzothiophene (2,8-DMDBT) [2]. In addition, some gaseous sulfur-containing moieties can be found, mainly H_2_S, SO_2_, and SO_3_, produced after burning or degradation of thiophenic compounds [2].

Sulfur oxides, especially SO_2_, which is the dominant oxide, are emitted in the environment upon combustion of S-containing fuels and can cause dangerous effects on health and the environment. The emitted SO_2_ can react with rainwater or air moisture and cause acid rain that can be transferred to soils, destroy foliage, cause corrosion of historical buildings, and decrease the pH of water bodies [3]. Besides, SO_2_ is known to have poisoning effects on the cars’ catalysts (TWC) due to the sulfates produced by sulfur-containing fuel, which lowers the catalyst efficiency. Sulfate aerosol particles formation, at a diameter of around 2.5 μm, can also be responsible for respiratory illnesses since they are able to penetrate into the lungs [4].

In order to control and prevent SO_2_ emissions, international agreements have been established from 1979 [5]. USA, Canada, and the EU have developed regulations primarily for transport fuels since they are the prime source for most of the SO_2_ emission. In 1993, the Clean Air Act (CAA), the comprehensive federal law of USA that regulates air emissions from stationary and mobile sources, stated a limit of 0.5 g kg^−1^ for sulfur concentration in diesel oil, while in EU, the limits were set in 1998 at the levels of 0.35 and 0.05 g kg^−1^ for the years 2000 and 2005, respectively [6]. From 2006, new regulations in USA targeted to reduce the sulfur content of on-road diesel fuel and gasoline from 0.5 g kg^−1^ and 0.35 g kg^−1^ to 0.015 g kg^−1^ and 0.03 g kg^−1^, respectively, targeting a maximum sulfur content limit in diesel of 0.01 g kg^−1^ by 2010. In spite of these regulations, the SO_2_ emissions will continue to increase, especially due to countries such as China that still depend on coal to fulfill their high energy demands, thereby contributing to air pollution [7,8]. Hence, in order to prevent the generation of these hazardous contaminants (SCs), exploring and developing various highly efficient, economical, and environmentally friendly methods is required.

## 2. Desulfurization Methods

There are generally two different approaches for eliminating SO_x_ emissions: precombustion and postcombustion treatment methods. The precombustion treatment method is applicable in the case of flue gas treatment and reduction of the SO_x_ emissions and of the sulfur present in the fossil fuel [9]. However, it is not a viable method due to the use of hot and corrosive effluents, the generation of carbon dioxide (CO_2_), and the produced refractory organic sulfur that is difficult to remove. For all these reasons, it is essential for additional methods to be developed, that can decrease the operation cost, minimize CO_2_ emission, and be feasible for the removal of the refractory part even under extremely invasive conditions. The main methods that have been developed for the desulfurization of fuels besides hydrodesulfurization (HDS) [10,11,12,13] include oxidative desulfurization (ODS) [12,13,14], biodesulfurization (BDS) [15], extractive desulfurization (EDS) [16], and adsorptive desulfurization (ADS) [17]. Among them, ODS [13,14] and BDS [15], present advantages that ingrain on the fact that by their application, fuel sulfur is removed under ambient conditions based on the property of organic sulfur compounds to form oxidized products that can be extracted.

The HDS process is the most widely used industrial desulfurization method [16], in which sulfur containing compounds (SCCs) are hydrogenated to H_2_S for the ease of separation. On the contrary, this method is not efficient for the elimination of aromatic SCCs, such as thiophenes and their derivatives, and only a minimum limit of 50 ppm of sulfur content can be removed [17,18,19]. During the operation, high temperature, pressure, and hydrogen are also required [20].

With the ODS process, the removable amount of SCCs can reach an ultralow level [13,14,21,22,23]. Since SCCs and their oxidized counterparts (i.e., sulfones and sulfoxides) are polar, they can be selectively removed after oxidation. During the ODS process, initially, SCCs with the aid of oxidizing agent are transformed to sulfones and sulfoxides and then, these oxidation products are extracted by a solvent. The drawbacks of this method are: (a) the fact that it is a multistep process, (b) the extraction part consumes energy, and (c) the use of oxidizing agents that may be corrosive or hazardous [13,21,23].

In the EDS process, the removal of SCCs is due to the higher solubility of the compounds in some solvents compared to hydrocarbons [16]. In addition, the selective removal by solvent extraction can be performed multiple times until the desired level of desulfurization is achieved. EDS can be performed at ambient conditions, resulting in lower consumption of energy. However, the use of expensive and nongreen solvents and the need for regeneration stages are the major drawbacks of this method [16].

Adsorptive Desulfurization (ADS) is an important method based on liquid-phase adsorption applied for ultralow-level desulfurization with important advantages, such as ambient operating conditions (near to ambient temperatures and atmospheric pressure) without the use of oxygen or hydrogen. Since ADS mainly depends on the adsorptive capacity of the material, the selection of the adsorbent is crucial. The main qualities for an effective adsorbent include a simple synthesis route, adsorption at ambient conditions, high porosity, regeneration capability, and low environmental footprint. The removal of SCCs from fuels using adsorbents has been successfully tried, and some of the best performing and promising materials include materials such as activated carbons (ACs) [24,25,26,27,28,29,30,31,32,33], zeolites [34,35,36,37,38,39], mesoporous silica, alumina and related materials [40,41,42,43,44,45], and ion exchange resins [46,47].

Recently, metal-organic frameworks (MOFs) have been stated to be a new category of prosperous materials that can be utilized as adsorbents for removal of SCCs. In this review, the main focus is to showcase the up-to-date research that has been carried out on the utilization of MOFs as sorbent materials in adsorptive desulfurization (ADS). Besides, the functionalization of these materials with their linkers, as well as the adsorption mechanisms that are proposed, are also discussed in order to illustrate the chemistry involved using MOFs during ADS.

## 3. Metal-Organic Frameworks (MOFs) as Efficient Adsorbents for Desulfurization

There has been a significant progress in the development of novel porous materials during the last few decades due to the rise in the importance of research in the field of materials science [42,43]. Among various new materials designed and synthesized during the past few years, metal-organic frameworks (MOFs) have been found to be promising candidates for a wide range of applications [48,49,50,51], due to high porosity, high surface area, and availability of active sites. In general, a MOF can be regarded as a coordination network of organic ligands and metal ion or metal clysters, containing potential voids, with one-, two-, or three-dimensional extended structures [52,53]. They consist of an inorganic center that can be either metal ions, a cluster of metal ions, or, in more advanced cases, a multinuclear complex. These inorganic centers, referred to as metal clusters/subunits or secondary building units (SBUs), are coordinated/linked each other via di- or poly-dentate chelating organic bridges/molecules, called linkers. Some typical linkers are benzenetricarboxylic acid (BTC), benzenedicar-boxylic acid (BDC) or imidazole. During MOF synthesis, the main template is the solvent, which has weak interactions with the framework, an important factor for obtaining products with neutral frameworks and accessible pores [54,55].

These hybrid inorganic-organic framework materials are known for their very high adsorption capacity from gaseous or liquid phases, with a characteristic paradigm of hydrogen adsorption/storage at moderate operation conditions. Even though the possibility to synthesize solid highly-porous materials based on coordination between metal ions and organic linkers was a well-explored research topic, in 1995, Yaghi and Li reported a hydrothermal protocol to obtain, via polymeric coordination between copper with 4,4′-bipyridine and nitrate ions, a “zeolite-like” crystalline structure [56]. Since it was a new class of hybrid materials, different names were proposed that are still in use [54,55], such as porous coordination networks [57], porous coordination polymers [58], microporous coordination polymers [59], zeolite-like MOFs [60], and isoreticular MOFs [61].

**Figure 2:**
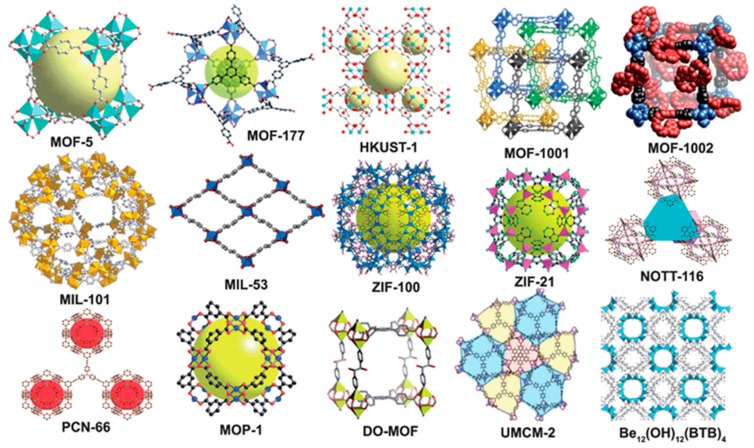
Metal-organic framework (MOF) structures (reproduced from [49] with permission from the Royal Society of Chemistry).

Due to the wide availability of potential metals and linkers, the number of possible structures of MOFs is virtually infinite. Some of the characteristic MOFs are collected in Figure 2. Interestingly, different frameworks can be developed since many metals of the periodic table can be involved, which can be in their singlet form or in the form of clusters, and various organic compounds can be utilized as linkers. For these reasons, MOFs can present a variety of physical and chemical properties, making them important materials. Clearly these features establish MOFs as promising materials for gas storage [62,63,64], separation of chemicals [54,65,66], catalysis [67], drug delivery [68], polymerization [69], magnetism [70], luminescence [71], reactive detoxification of toxic compounds [72], and especially adsorption [55,73], including sulfur containing compounds (SCCs) [74], due to their properties, i.e., large pore volume and high surface area [75,76].

## 4. Desulfurization with MOFs

Fluid catalytic cracking (FCC)-obtained naphtha [77] contains sulfur content on the order of 200−7000 ppmw, while the large majority of the sulfur content of the gasoline originates from FCC naphtha. FCC naphtha contains hydrogen sulfide, thiols, disulfide, thiophene, and its alkyl derivatives, with the two latest representing 60−70 wt% of the total sulfur compounds [78,79]. The adsorption process to remove thiophene and alkylthiophenes in FCC naphtha [80,81] has to be highly selective for adsorption of thiophenic molecules versus the major components of FCC naphtha, that is, paraffins (20%−40%), naphthenes (5%−15%), olefins (20%−40%), and aromatics (20%−40%). Thiophene (TP), 3-methylthiophene (3-MT) and 2,5-dimethylthiophene (2,5-DMT), in the order of 2,5-dimethylthiophene < 3-methylthiophene < thiophene < benzothiophene, were found to be adsorbed on Cu^+^-13X zeolites [82]. The potential of MOFs for being successful sulfur-selective adsorbents for thiophenic molecules from model feed was reported for HKUST-1, CPO-27-Ni, RHO-ZMOF, ZIF-8, and ZIF-76 by Perlada et al. [77]. Besides, four different MOFs consisting of two different metals (Cu^2+^ and Cr^3+^) proved to be promising adsorbents for 3-methylthiophene (3-MT) from model oil [83]. A double adsorption mechanism by physisorption and chemisorption was proposed as the main mechanism [83]. HKUST-1 (or Cu-BTC) was also examined for thiophene and tetrahydrothiophene (THT) adsorption, achieving 78 wt% sulfur content removal from thiophene-containing model oils [84]. An even higher removal of up to 86 wt% was obtained for THT-containing model oils [84]. Three conjugated polycarbazole porous organic frameworks, named o-Cz-POF, m-Cz-POF, and p-Cz-POF, that possessed ortho, meta, and para steric configuration, were also examined for adsorption of 3-methylthiophene [85]. The highest uptake amount of 3-methylthiophene was observed in m-Cz-POF, which could reach 7.762 mmol/g (248.4 mg of S/g) at 298 K. This value is far beyond those of the porous absorbents previously reported [85]. Various MOFs have been used to selectively adsorb organo-sulfur compounds [59,86]. The first reported work highlighting the use of MOFs as adsorbents for adsorptive desulfurization (ADS) was carried out by the research group of Matzger in 2008, where the removal of BT, DBT, and DMDBT was successfully carried out using various MOFs such as HKUST-1 (also known as Cu-BTC), UMCM-150, MOF-5, MOF-505, and MOF-177 [41]. During the adsorption, MOFs interact with S-compounds present in the fuel predominately by π–π interactions [40,41]. Moreover, they also develop metal-S coordination bonds through unsaturated coordination sites of selected metal ions such as Cu^2+^, Zn^2+^, Co^2+^, Ni^2+^, and Cu^+^ [41]. Some of the other MOFs that have been reported to be highly efficient for ADS are: HKUST-1 [22,47,87], UMCM-152 [88], CuCl/MIL-47(V) [89], MIL-101(Cr) [89] MIL-100(Fe)], MOF-505 [84], PWA/HKUST-1 [90], and Cu_2_O/MIL-100(Fe) [74].

In the work carried out by Matzger and coworkers [20,59,91], the maximum ADS capacities were reported to be 0.38 mmol/g (51 mg/g) for BT using MOF-5 while using UMCM-150 the adsorption capacity was 0.45 mmol/g (83 mg/g) and 0.19 mmol/g (35 mg/g) for DBT and DMDBT, respectively. In addition, the maximum adsorption capacities were reported to be higher than those presented by Na-Y zeolite [37,88]. Commonly, the high surface area and pore volume of the adsorbent is stated to be the main reason for a good adsorption. However, Matzger was able to show that the adsorption studies using MOFs showed opposite trend, indicating that the porosity of MOFs is not the key governing factor for SCCs’ adsorption. MOF-177, which had the highest porosity among the materials tested in his study, showed the lowest maximum adsorption capacity, thereby implying the fact that the chemical properties of the active sites are much more important.

Similarly, adsorption studies of aromatic sulfur compounds have been investigated by various research groups with different types of MOFs for obtaining low-sulfur liquid fuels [58,77,87,92,93,94,95,96,97,98]. A list of ADS results in the liquid phase using different MOFs is presented for the adsorption of benzothiophene (BT) in Table 1 and the adsorption of dibenzothiophene (DBT) in Table 2. In Table 3, adsorption of BT, DBT, and 4,6-dimethyldibenzothiophene (4,6-DMDBT) using different MOFs for varying experimental conditions are shown.

HKUST-1, which is one of the most influential frameworks presented in 1999 by Chui et al., is assumed as a benchmark MOF, especially for gaseous adsorption-oriented applications. Its secondary building unit (SBU) consists of a paddle wheel shaped metal cluster of Cu_2_(CO_2_)_4_ that is formed by a dimer of copper ions, with each Cu^2+^ ion being coordinated with four benzene-1,3,5-tricarboxylic acid (BTC) groups, thereby acting as a tritopic linker. The adsorption isotherms and capacities of HKUST-1 for BT, DBT, and 4,6-DMDBT from iso-octane were studied using batch experiments at room temperature [66]. For an initial sulfur content of 1500 ppmw in the model fuel, the adsorption capacity was found to be 25 g S/kg sorbent for BT, while for DBT, the capacity reached 45 g S/kg sorbent. In the case of adsorption of 4,6-DMDBT from a sulfur content of 600 ppmw S in the model fuel, the adsorption capacity was found to be 16 g S/kg of sorbent. Similar studies for adsorption of BT, DBT, and 4,6- DMDBT using C300 Basolite MOF (HKUST-1 commercially available and produced by BASF) in iso-octane have been carried out [76] for an equilibrium time of 72 h at 304 K. For initial concentration of 1724 ppmw, the adsorption capacity was found to be 40 g S/kg for BT, 45 g S/kg for DBT and 13 g S/kg for 4,6-DMDBT. In another study using the same MOF, C300 Basolite, the adsorption capacity of BT, and DBT, in iso-octane after 24 h, for an initial concentration of 370 ppmw S, was found to be 81 g S/kg for BT and 32 g S/kg for DBT [76]. MOF-199 was also examined as an adsorbent for the removal of DBT in dodecane, as the model fuel. For an initial DBT concentration of 50 ppmw and a dosage of 5 wt% of MOF-199, the final DBT concentration in the outlet of the two-stage hydrocyclones was 8.79 ppmw with a separation efficiency as high as 99.75% within 30 s [99]. Similarly, MOF-14 was used for the removal of BT, DBT, and 4,6-DMDBT. The experimental results indicated that MOF-14 possesses high selectivity for the organosulphur compounds, a characteristic feature that was not found for other adsorbents [106].

## 5. Functionalization of MOFs

MOFs can be upgraded using various modification techniques such as grafting, impregnation, addition of functional groups at the linkers, or making composites materials [107,108,109,110]. Important advances were made in obtaining more complex structures having higher order of structures using nanocrystals of MOFs as building units [111]. Functional materials can also be grafted to the Lewis acid CUSs of the MOFs. As discussed above, the importance of the chemical features was proposed by the research group of Matzger, who reported that adsorption of SCCs was highly correlated with the functional groups rather than the porosity of MOFs. Metal salts, CuCl_2,_ Cu_2_O, γ-Al_2_O_3_, heteropolyacids, different MOFs, pyrazine, NH_2_ and SO_3_H groups, graphite or graphite oxide, or are some of the functionalities reported in the literature, and some of them are collected and presented in Table 4.

Metal salts presenting Lewis acidity have been proven to enhance the adsorption of basic contaminants after being impregnated on a MOF surface. Optimization of the impregnation is always needed in order to overcome the decrease in porosity upon modifications, which can have impact on their adsorption capacity. A CuCl_2_-loaded vanadium terephthalate framework (MIL-47) presented an increase of the adsorption capacity for the adsorption of benzothiophene (BT) from n-octane by 122% compared to the pristine MIL-47. The increase could be due to the π-complexation mechanism between the thiophene ring of the BT molecule and Cu(I) [74].

However, the MIL-53s (Al and Cr) loaded with CuCl_2_ did not present improvement on the adsorption of BT [90]. Unlike V(III) of MIL-47, Al(III) and Cr(III) were not capable of the reduction of Cu(II) to Cu(I). Cu(I) species in the Cu_2_O-loaded MIL-100(Fe) and MIL-101(Cr) [101] were used for the adsorption of BT from n-octane. The presence of Cu(I) species in the porous network of MIL100(Fe) decreased the porosity by 9% but showed a 16% increase in the adsorption capacity compared to initial MIL-100(Fe). The formation of π-complexes during the adsorption of SCCs contributed to the higher adsorption capacity of the metal loaded MOF than the virgin ones [101]. 

A bimetallic MOF (Zn/Cu-1,3,5- benzenetricarboxylate (BTC)) was examined for the adsorption of DBT by Wang et al. [105]. The bimetallic MOF presented an increase in the adsorption capacity for DBT than the virgin Cu-BTC due to the interaction of the Zn(II) π-complex with the π-electrons of DBT [105]. Hasan et al. reported the adsorption of BT and DBT adsorption from liquid fuel using a composite of two different MOFs via π-complexation [101]. In another work, the surface acidity of a MOF was enhanced by using heteropolyacids (HPAs) [101]. These strategies led to an enhance removal of basic SCCs. Huang et al. functionalized MIL-101(Cr) (chromium terephthalate) with –SO_3_H groups to form AgO_3_S-MIL-101(Cr), and it was further used for BT and DBT adsorption from liquid fuel [97].

Pristine and functionalized UiO-66 (Zr) was also tried for removal of thiophene (TP) and benzothiophene (BT). The functionalization involved the introduction of amino (–NH_2_) groups at the linker and introduced carboxylic (–COOH) groups at or as the defectous sites. Even though the functionalized MOFs presented decreased porosity compared to the pristine one, they showed increased adsorption capacity. The authors linked this effect to the hydrogen bond sites in their surface as well as acid–base interactions [104]. MOF-74(Ni) was impregnated on γ-Al_2_O_3_ beads for the synthesis of MOF-74(Ni)@γ-Al_2_O_3_ composite [114,115], which showed excellent DBT and BT adsorption. This enhanced adsorption was attributed to strong metal-S bonding between the adsorbent and SCCs [102]. Similarly, using an in situ green synthesis method, a composite, Al(OH)(1,4-NDC)@γ-AlOOH, was prepared from 1,4-H_2_NDC (1,4-naphthalene dicarboxylic acid) and porous γ-Al_2_O_3_ beads and was tried for the adsorption of SCCs. The composite presented maximum adsorption capacities with the following order: benzothiophene > dibenzothiophene > 4,6-dimethyldibenzothiophene > thiophene. The main adsorption mechanism was due to the presence of Lewis acid sites on the metal (Al) [112]. 

Composites of metal organic frameworks (HKUST-1) with graphite oxide (GO) were also reported to be efficient adsorbents. With a minimal content of GO (~1.75%), the composite MOF (GO/HKUST-1) showed sufficient desulfurization results for TP (adsorption capacity 60.67 mg S/g) that were attributed to the improved porosity [118]. With the green solvothermal method, a MOF (Cu-BTC) and a MOF/Graphene (Gr) hybrid nanocomposite were also prepared and used as adsorbents for DBT removal. The experimental results showed that MOF/Gr (9:1 wt ratio) presented a high dibenzothiophene adsorption capacity for DBT, 46.2 mg S/g, compared to the unmodified MOF sample that presented an adsorption capacity of 35 mg S/g [119].

Metal organic frameworks decorated on fabric composites, (MOF)@fabric, such as MIL-53(Al)-NH_2_ in-situ prepared within fabrics (cotton or/and wool), have been used for thiophene adsorption from n-heptane [120]. The Q_max_ followed the order of MIL-53(Al)-NH_2_ (739.0 mg/g) > MIL-53(Al)-NH_2_@fabric (469.4–516.5 mg/g) >>> fabric (83.1–153.8 mg/g) [120].

## 6. Mechanisms of Desulfurization

**Figure 3:**
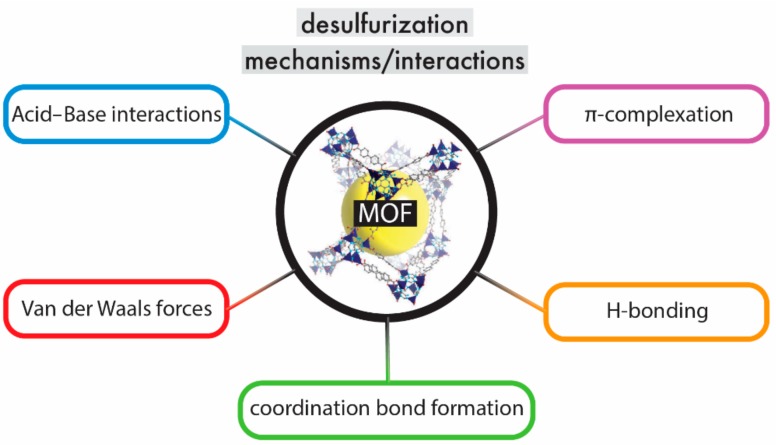
The predominant desulfurization interactions/mechanisms of MOFs.

Adsorptive desulfurization has been attributed to different mechanisms/interactions. The main adsorption mechanisms are collected in Figure 3, include the acid–base interactions (Lewis acid–base), coordination bond formation, π–π complexation, Van der Waals force, and H-bonding [113,121,122].

### 6.1. Effect of Porosity

The porosity of the adsorbents plays a crucial role in adsorption because it influences their adsorption capability. Moreover, during adsorptive desulfurization, there is the opportunity of selective separation of molecules that have different molecular size. This is true when the molecular size of the thiophenic derivative is smaller than the MOF pores. During adsorption, molecules can diffuse into the porous channels and become anchored at the active adsorption sites [92]. On the contrary, if the molecular size is similar or smaller than the pore sizes of the MOF, steric hindrances forbid the penetration inside the framework and adsorption cannot take place [122].

### 6.2. Acid–Base Interactions

Acid–base interactions are the most common mechanism involved in ADS. Many MOFs can act as Lewis acids due to coordinatively unsaturated metal sites (CUSs) that are able to accept a pair of electrons by forming coordination bonds with molecules having a lone pair of electrons (Lewis acid sites) [123]. Hence, the adsorption of the majority of SCCs can be attributed to interactions with the Lewis acidic metal ion sites by coordination.

Thiophenic compounds, due to their solitary electrons, can be regarded as bases and thus can be easily adsorbed onto MOFs’ CUSs via acid–base interactions. Bases can be classified into polarizable and nonpolarizable, and after the Pearson’s hard and soft acid–base characterization, these types are denoted as “soft” and “hard” bases, respectively. Acids can also be classified as hard or soft based on their interactions with hard or soft bases. For example, soft SCC bases strongly interact with soft Lewis acids, such as Cu^2+^, Zn^2+^, and Co^2+^ [123].

### 6.3. Coordination Bond Formation (Lewis Acid–Base Interaction)

Several MOFs, such as HKUST-1, MOF-74 (Ni, Mn, Co, etc.), MIL-100(Cr, Fe), and MIL-101(Cr) [61,76,91,102,124,125] etc., have proved to be promising adsorbents due to the fact that their CUSs are surrounded by regular pore channels that can be used to induce region-selective interactions. This is not possible with adsorbents like zeolites, activated carbons, or mesoporous silica [126].

Adsorption of thiophenic compounds via hydrogen bonding has also been reported; however, this kind of bonding is not common in adsorption of SCCs. Voorde et al. studied the adsorption of heterocyclic SCCs by MIL-53(Fe) and reported that the adsorbates have the capability to form hydrogen bonds (as acceptor for hydrogen bonds) with MIL-53(Fe) [127].

### 6.4. π-Complexation

Some metal ions, such as Cu^2+^, Ag^+^, Pd^2+^, and Pt^2+^, have shown adsorption ability for SCCs through π-complex formation [89]. The complexes formed via electronic interaction between some metal cations and π-electron clouds of the chemicals, known as π-complexes [89]. Metals with empty s-orbitals can be π-complexed with the sulfur of the thiophenic compound, thereby creating a σ-bond [48]. Adsorbates with high π-electron densities (i.e., polyaromatic hydrocarbons) are greatly favorable for π-complexation. Adsorptive removal of SCCs by π-complexation was first reported in 2003 by Yang et al., in which adsorption studies where carried out by metal modified Y zeolites [19] and were described as “back-donation effects”. A weak interaction can also be created between electron-rich and electron-poor aromatic groups, leading to the aromatic compounds being adsorbed via π–π stacking. This is a widely occurring mechanism in aromatic systems but with limited selectivity, especially for ADS.

**Figure 4:**
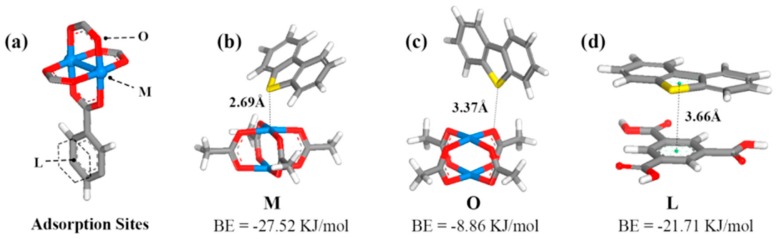
(**a**) Three adsorption sites (M-, O-, L-) on Cu-BTC. Adsorption configuration and BEs of DBT on (**b**) M-, (**c**) O-, and (**d**) L-sites of Cu-BTC. adapted with permission from [92]. Copyright (2014) American Chemical Society.

Based on computational and experimental results, Wu et al. studied the nature (sites, configuration, and energies) of the adsorption for thiophenic compounds over HKUST-1 [92]. The results derived from DFT calculation revealed three possible adsorption sites; via coordinative unsaturated copper sites from the cluster (M-site), via oxygen from the coordinated to copper carboxylic group (O-site), and via the phenyl part of the linker (L-site). The adsorption energy and configuration upon adsorption of DBT at the above-mentioned three sites are demonstrated in Figure 4. Adsorption at the L-sites is not feasible, because of the presence of the neighboring clusters and the bulky in size DBT, which is bigger than the linker and the space in between the clusters (8.0 Å between neighboring M-sites). In order to overcome these steric hindrances, a possible strategy is to increase the size of the linker, resulting in an increment of the distance and space between the metallic cluster, and based on this, the adsorption via the L-sites it will be feasible. More details of this are discussed herein after.

**Figure 5:**
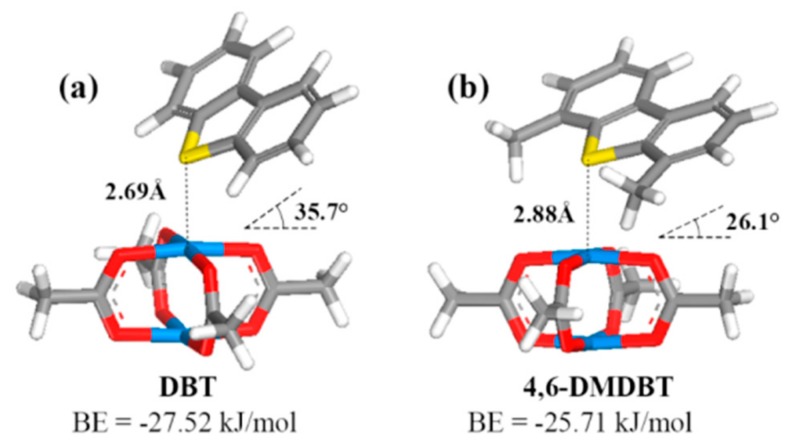
Adsorption configuration and energies of DBT and 4,6-DMDBT adsorption on Cu coordinatively unsaturated metal sites (CUS), adapted with permission from [92]. Copyright (2014) American Chemical Society.

The authors concluded that adsorption can take place only via the coordinatively unsaturated metal sites (CUS) or M-sites, as presented in Figure 4b, which is consistent to that reported previously for adsorption of H_2_O and CO_2_. The CSUs can interact with either the lone electron pair of sulfur atom (σ-M interaction) or the conjugated π systems of the two rings of DBT (π-M interaction). For 4,6-DMDBT, the presence of the alkyl groups increases the adsorbate’s electron density, which increases the π–M interaction. On the contrary, the alkyl groups introduce steric hindrance, which has a negative impact on the σ–M interaction. The comparison of the results obtained for the adsorption of DBT and 4,6-DMDBT from the DFT calculations can be seen in Figure 5.

Functionalities also play an important role in the π-complexation mechanism; π-electron-rich compounds with no functionalities have no adsorption ability since the adsorbent–adsorbate interactions (coordination, acid–base, and H-bonding) are difficult to occur. Among functional metals, Cu(I) functional sites, when π-complexed into MOF materials, increased their adsorption capacity. For example, CuCl_2_-loaded MIL-47 presented a higher adsorptive performance in the adsorption of benzothiophene (BT) from n-octane than the pristine MOF [20], due to a π-complex between Cu(I) sites and porous MIL-47, which resulted in a BT adsorption capacity of 122%. Cu(I) sites, when functionalized on MIL-101(Cr), MIL-100(Fe), and CuBTC, presented higher adsorbed amounts of SCCs than the virgin ones [91]. Cu_2_O-loaded MIL-100(Fe) introduced Cu(I) into the network of MIL100(Fe) and caused a 16% increase of the maximum adsorption capacity (Q_0_) compared to the initial MIL-100(Fe), although the porosity was decreased by 9% [76]. Dai et al., examined MOF-5-based π-complexing adsorbents with different concentrations of Cu(I) for the adsorption of DBT from n-octane at dynamic mode [89]. With the increase of Cu(I) content, the breakthrough and saturation sulfur capacity of the adsorbents increased from 2.11 and 5.05 wt% for MOF-5, respectively, to 5.89 and 8.59 wt% for 2 mmol of Cu(I) into 1 g of MOF-5 and to 9.42 and 10.94 wt% for 3 mmol of Cu(I) into 1 g of MOF-5.

### 6.5. Van der Waals Forces

Van der Waals interactions are generally very weak interactions in molecules and play an important role in adsorption, since they are only applicable at low temperatures. Besides, if no special chemical interactions are created between compounds, they are usually adsorbed through van der Waals forces. For MOFs, due to their high porosity, when other mechanisms are unfavored, adsorption can be achieved mainly by van der Waals forces [113]. Other interactions, such as electrostatic interactions, that have been frequently applied to explain contaminant removals during water purification have not been reported in ADS. This might be due to the low possibility for cationic or anionic SCCs.

## 7. Drawbacks

Apart from all the advantages, MOFs also have some drawbacks, which have to be considered during their application in ADS. MOFs are not very stable at high temperatures and for this reason they are not appropriate for applications at elevated temperatures. Additionally, some frameworks such as HKUST-1, are known to be sensitive to humidity, while others, such as UiO-66, are stable. Detailed analysis of the stability of MOF structure after synthesis and utilization is crucial for maintaining higher adsorption and selectivity of SCCs during adsorptive desulfurization. Synthesized pristine MOF materials are usually in fine powder form with poor mechanical strength, making it difficult for recycling and reuse in actual operation. Another important drawback is particle aggregation, which may take place during the process of adsorption and recycling treatment, leading to decreased accessibility of the reactive sites, and consequently diminishing the adsorbent activity. Finally, the small pore apertures of many MOFs cause high diffusion resistance due to steric and dynamic hindrance, thus restricting movement of S-containing molecules into the pores and thereby resulting in adsorption on the surface rather inside the framework. This may result in low utilization of unsaturated metal sites, surface area, and, ultimately, the adsorption capacity. In catalysis, the problem of fast deactivation of the catalyst due to incomplete desorption of reaction products is well understood. Similarly, regeneration of “spent” sorbent is of importance, since its efficiency determines the usable lifetime of the sorbent, operating costs, and practical aspects of the scale-up of the desulfurization process.

Although there are drawbacks, the utilization of MOFs as adsorbents is of great importance due to the fact that adsorption is performed at mild conditions, and, at these conditions, MOFs can be successfully used for adsorptive removal due to their large porosity and great functionalization properties.

## 8. Perspectives

**Figure 6:**
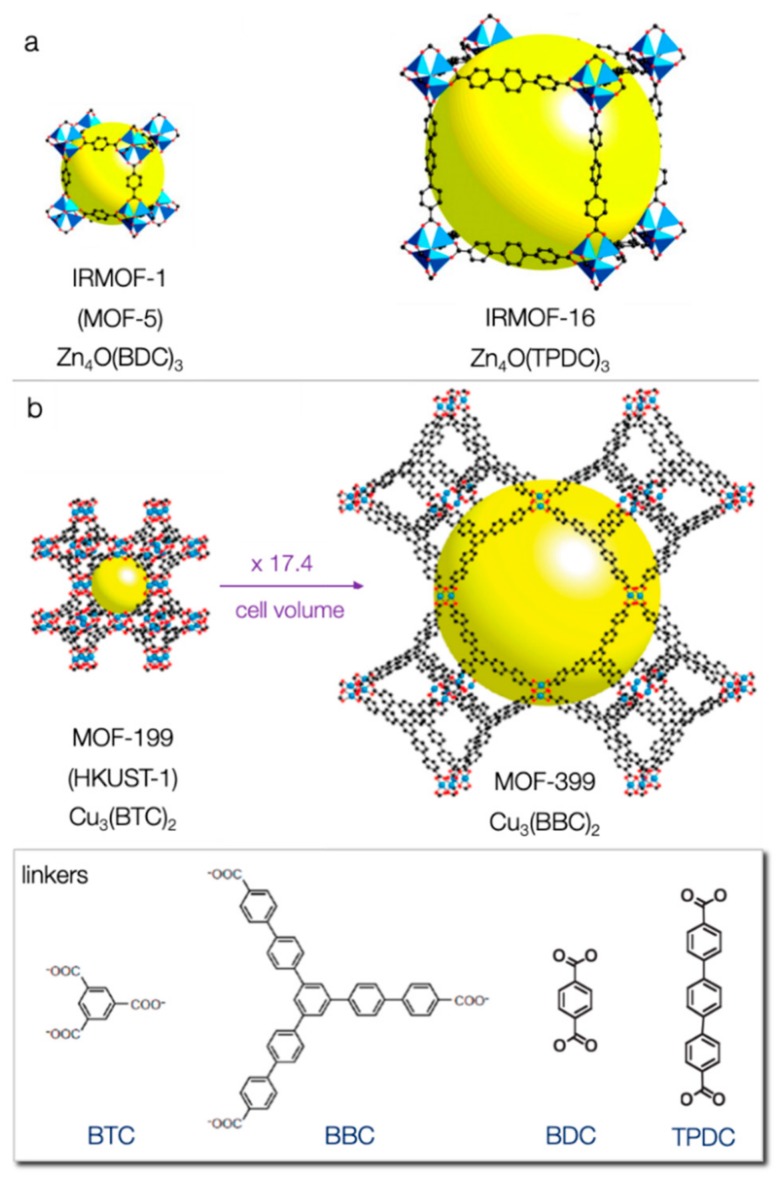
A scaled comparison of the single crystal structure of (**a**) MOF-5 and (**b**) HKUST-1 and the largest representatives of their isoreticular family (the yellow spheres represent the maximum volume of the biggest cavity of each structure (**a**: adapted with permission from [61]. Copyright (2002) The American Association for the Advancement of Science, **b**: adapted with permission from [48]. Copyright (2011) American Chemical Society).

Expect the above-mentioned drawbacks that should be explored and be overwhelmed, a great challenge is to enhance the adsorptive desulfurization capability from fuels by MOFs. In this direction, the most crucial aspect is the enhancement of the availability and density of the coordinatively unsaturated metal sites (CUS). Increase of the porosity and, more importantly, the aperture and size of the pores will lead to beneficial effects. A followed strategy in order to enhance the surface area and pores volume is the use of longer organic linkers in order to expand the structure, while the underlying topology (structure/net of the framework) remains the same [48,61,119,128,129,130,131,132] A typical example is the exchange of the bicarboxylate linker BDC^2−^ with TPDC^2−^ (terphenyl-4,4′′-dicarboxylate), as can be seen in Figure 6a. The longer linker leads to a bigger unit cell edge and cages/pores. These two MOFs, as well as all of this family, are called isoreticular (IR) and have the same primitive cubic net shape. MOF-5, which is the parent framework of this isoreticular family, is abbreviated as IRMOF-1, while the one with TPDC^2−^ is abbreviated as IRMOF-16. A smaller isoreticular to MOF-5 is Zn_4_O(fumarate)_3_ (furamate: ^-^OOCCH=CHCOO^-^), reported in 2009 by Xue et al. [61]. The latter has half the unit cell edge and the volume of the cage is decreased by eight-fold compared to IRMOF-16, while the surface area is almost half of that of MOF-5 (1120 m^2^/g BET surface area) [131,132]. The theoretically geometrically calculated (by Monte Carlo integration approach) surface area of IRMOF-16 was reported as 6074 m^2^ g^−1^ [130]. The experimentally calculated BET surface area for this MOF was reported to be between 472 and 1912 m^2^ g^−1^, depending on the solvent evacuation/activation process. This can be linked also to the instability of the structure upon exposure to humidity (collapse of interparticle voids), and, for that reason, experimental research regarding this series of isoreticular MOFs is limited [130]. HKUST-1 or MOF-199 is the smallest member of its isoreticular family, while the largest member is Cu_3_(BBC)_2_ or MOF-399, as shown in Figure 6b. The latter has a 17.4-fold larger cell volume than HKUST-1. It is worth mentioning that MOF-399 has the lowest density (0.13 g cm^−3^) and greatest void fraction (94%) reported of any MOF to date [48,131]. These values are 0.88 g cm^−3^ and 72%, respectively, for HKUST-1.

**Figure 7:**
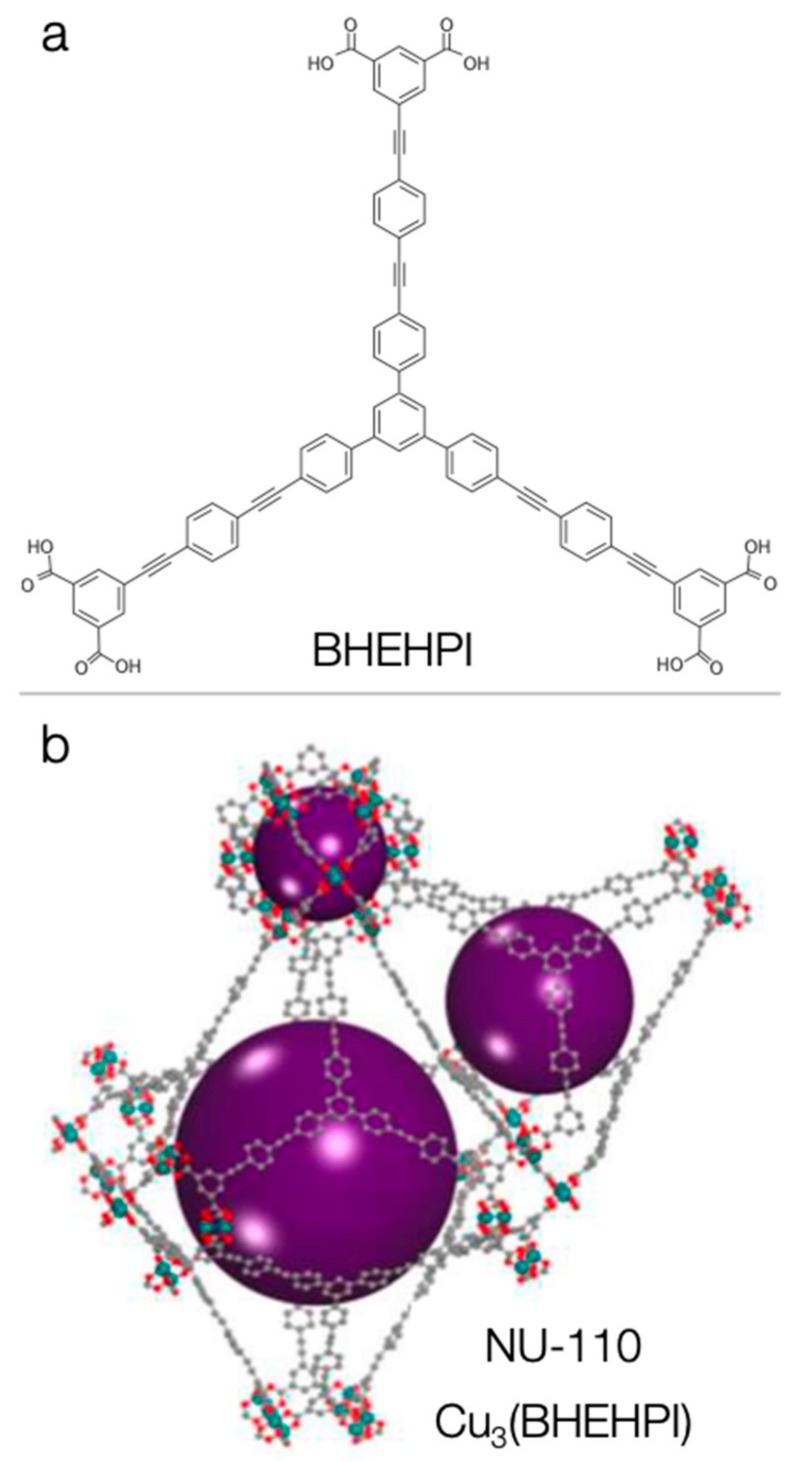
(**a**) The chemical structure of the ligand and (**b**) the different cages of the NU-110 framework. Adapted with permission from [132]. Copyright (2012) American Chemical Society.

The expansion of the linker with alkyne rather than only phenylene units led to an even higher increase of surface area and total pore volume. The characteristic example is Cu_3_(BHEHPI) or NU-110 (NU stands for Northwestern University in Chicago, USA), which has the highest reported surface area and total pore volume up to date [132]. This copper-based MOF with a hexacarboxylate macromolecule as a ligand (BHEHPI^6–^ stands for 5,5′,5′′-((((benzene-1,3,5-triyltris(benzene-4,1-diyl)) tris(ethyne-2,1-diyl))-tris(benzene-4,1-diyl)) tris(ethyne-2,1-diyl)) triisophthalate) was reported by O. Farha, J. Hupp and coworkers in 2012 and is shown in Figure 7 [132]. Using the N_2_ sorption experiments, the ligand-modified Cu-MOF revealed a BET surface area of 7140 m^2^g^−1^ and a total pore volume of 4.4 cm^3^g^−1^. Interestingly, the obtained nitrogen isotherm was closer to type IV rather than type I, revealing multiple sizes of pores, a fact which is consistent with the different types of cages illustrated in Figure 7. The authors showed also that the MOF’s theoretical surface area can reach up to 14,600 m^2^ g^−1^ [132].

Another strategy for the upgrading of MOF desulfurization performance is the formation of nanocomposites with metal-free fillers such as graphite (GR), graphite oxide (GO), graphitic carbon nitride (g-C_3_N_4_), or oxidized graphitic carbon nitride nanospheres (g-CNOx) [133,134,135,136,137,138,139]. Petit, Bandosz, and coworkers showed that the addition of a limited amount of GO (5 wt% of the final composite’s mass) during the synthesis of HKUST-1 led to an enhancement of the hydrogen sulfide adsorption capacity by more than two-fold compared to pure HKUST-1, reaching a very high capacity of almost 200 mg/g [134,135,136,137]. The composite formation led to a wide range of positive aspects, such as increment of the porosity and surface chemistry heterogeneity, improved dispersion, density, and availability of the active adsorption sites, redox reactivity, and more [133,134]. The interactions were linked to the interactions of sulfur with the copper of the cluster, as was also reported in the case of copper hydroxide/oxide [137], while the addition of GO increases their availability through the formed defects effect. Ahmned et al. synthesized a highly porous MOF composite, consisting of Cr-benzenedicaboxyate and graphite oxide [115]. The addition of the GO resulted in an increase of the porosity, which had a positive effect on the removal of nitrogen- and sulfur-containing compounds from model fuel. Chen et al. reported that HKUST-1 composite with GO (1.75 wt%) has 61% increased adsorption capacity against thiophene (0.72 mmol/g) compared to virgin HKUST-1 [139].

**Figure 8:**
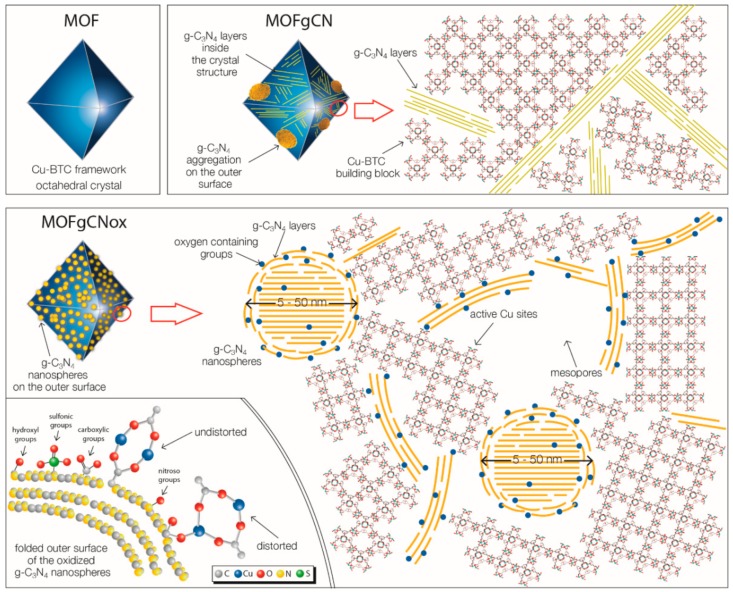
A schematic illustration of the HKUST-1-based nanocomposite with nanospheres of oxidized graphitic carbon nitride as filler. Reproduced from [143] with permission from the Wiley.

Various reports have shown that the incorporation of g-C_3_N_4_ inside the MOF matrix leads to elevation of the removal and reactivity capabilities of the composites compared to the pristine MOF [140,141,142]. Going a step further, Bandosz and co-workers synthesized a HKUST-1-based nanocomposite with nanospheres of oxidized graphitic carbon nitride (gCNox) [143,144,145]. The latter were incorporated inside the matrix of the framework and were dispersed on the outer surface of each particle. Due to the enhanced chemistry heterogeneity, the gCNox acted as linkers, predominately via the carboxylic groups. The formed nanocomposites revealed dramatic alterations of the optical, structural, textural, and chemical features, while formation of mesoporosity was also determined. The proposed illustration of the composite structure can be seen in Figure 8. This MOF-based composite showed significantly higher adsorptive and catalytic reactivity compared to pristine MOF, which was linked to the enhancement of the uncoordinated active copper sites’ availability and the formation of defectous sites in the framework. UiO-66 based composite with gCNox was also reported [145]. The growth of the framework in this case was around these nanospheres, with the final composite materials possessing higher catalytic activity compared to pristine UiO-66.

## 9. Conclusions

Adsorptive desulfurization (ADS) presents many advantages for application in fuel purification/desulfurization. The main advantages are that the ADS can be performed at ambient conditions of pressure and temperature, as well as the non-requirement of hazardous additives/chemicals, such as hydrogen. Since thiophene derivatives are difficult to remove by hydrodesulfurization (HDS), ADS present a promising alternative procedure for ultradeep desulfurization. Among the different adsorbents, MOFs have been demonstrated as a promising new class of materials for deep desulfurization applications due to high ADS capabilities. The current results reveal that the selectivity and adsorption capacity are further enhanced after functionalization of the MOF surface. Sulfur compounds can diffuse into the MOF’s channels and can be adsorbed into the MOF’s pore system via π- complexation, acid–base interactions, etc. The promising application of MOFs for adsorption-based desulfurization is expected to increase their use in industry. For the elimination of the drawbacks and for elevating MOF performance, further research of isoreticular MOFs with larger linkers, and of composite formation should be performed.

## Figures and Tables

**Table 1:** MOFs as adsorbents for benzothiophene (BT).

Adsorbent	Conditions or Remarks	Adsorption Capacity (mmol/g)	Ref.
MIL-53(Cr)	*n*-Octane solvent, 298 K	0.60	[89]
MIL-53(Al)		0.26	[89]
MIL-47(V)		1.6	[89]
NENU-511	*i*-Octane solvent, 298 K	2.2	[99]
NENU-512	*n*-octane	1.4	[99]
NENU-513		1.1	[99]
NENU-514		1.0	[99]
Zr(BTC)	liquid fuel	290 mg/g	[100]
ZIF-8	*n-*octane	45	[101]
MIL-100(Fe)		114	[101]
MIL-101(Cr)		35.77%	[92]
MIL-100(Fe)		20.76%	[92]
MOF-74(Ni) MIL-101		76.97 36.4	[102] [103]
UiO-66		19.83	[104]
HKUST-1		18.2	[92]

**Table 2:** MOFs as adsorbents for dibenzothiophene (DBT).

Adsorbent	Conditions or Remarks Solvent, Temperature (K)	Adsorption Capacity	Ref.
NENU-511	*i*-Octane	2.6 mmol/g	[99]
NENU-512		2.2 mmol/g	[99]
NENU-513		2.0 mmol/g	[99]
NENU-514		1.9 mmol/g	[99]
HKUST-1		7.7 mgS/g	[92]
MIL-101(Cr)	*n*-octane	32.5 mgS/g	[92]
ZIF-8		45 mgS/g	[101]
MIL-100(Fe)		114 mgS/g	[101]
MOF-101		52.4 mg/g	[101]
MIL-100(Fe)		35.77%	[101]
MIL-101(Cr)		20.76%	[101]
MOF-74(Ni)		85.05%	[102]
MOF-505		39.2%	[91]
MOF-199	dodecane	90%	[105]

**Table 3:** MOFs as adsorbents for BT, DBT, and 4,6-dimethyldibenzothiophene (4,6-DMDBT).

Adsorbent	Adsorbate (SCC)	Conditions or Remarks Solvent, Temperature (K)	Adsorption Capacity (mmol/g)	Ref.
UMCM-152	DBT/DMDBT	*i*-Octane, 298 K	1.8, 2.6	[38]
UMCM-153			2.8, 1.2	[38]
MIL-101(Cr)		Octane, 298 K	0.20/0.17	[65]
MIL-100(Fe)			0.20/0.25	[65]
HKUST-1			0.57/0.28	[65]
MOF-505	BT/DBT/DMDBT	*i*-Octane, 298 K	0.38/0.21/0.13	[91]
UMCM-150			0.30/0.45/0.19	[65]
HKUST-1			0.19/0.24/0.08	[65]

**Table 4:** Functionalized MOFs as adsorbents for adsorptive desulfurization (ADS).

Adsorbent	Functionalizing Group	Adsorbate (SCC)	Solvent	Adsorption Capacity (mgS/g)	Ref.
MIL-53(Al)	Al	BD	n-octane	8.3	[112,113]
MIL-53(Cr)	Cr			23.6	[112,113]
IL/MIL-101(Cr)	Cr			0.65	[112,113]
MIL-101	-			36.4	[102]
Cu/MIL-101	Cu			52.0	[102]
Ce/MIL-101	Ce			45.6	[102]
Cu-Ce/MIL-101	Cu-Ce			62.1	[102]
Cu-MIL-100-Fe	Cu			-	[101]
Cu_2_O/MIL-100(Fe)	Cu_2_O			1.1	[101]
CuCl/MIL-47(V)	CuCl			2.3	[74]
MOF-74(Ni)@γ-Al_2_O_3_	γ-Al_2_O_3_			87.77	[102]
UiO-66-NH_2_	–NH_2_			-	[104]
UiO-66-COOH	–COOH			22.6	[104]
HPA/IL@ZIF-8	HPA			68	[101]
HPA/IL@MIL-100(Fe)	HPA			167	[101]
PWA/HKUST-1	PWA			1.1	[110]
HPW(1.5)/Zr(BTC)	HPW		liquid fuel	238	[100]
Al(OH)(1,4-NDC)@γ-AlOOH	γ-AlOOH			-	[112]
MIL-101(Cr)-SO_3_H	–SO_3_H	BT, DBT	n-octane	9.95, 2.14	[92]
MIL-101(Cr)- SO_3_Ag	–SO_3_Ag			28.8, 31	[92]
MIL-101(Cr)-NH_2_	–NH_2_			2.6, 5.6	[97]
MIL-101(Cr)-NO_2_	–NO_2_			1.2, 2.1	[97]
Ag^+^/MOF-101(L)	Ag^+^	DBT		50.9	[92]
Ag^+^/MOF-101(M)	Ag^+^			47.8	[92]
Ag^+^/MOF-101(H)	Ag^+^			42.7	[92]
Cu-BTC/Gr				46.2	[105]
CuCl/MOF-5	CuCl			3.4	[105]
MOF-74(Ni)@-γAl_2_O_3_	γ-Al_2_O_3_			76.97%	[114,115]
MOF-74(Ni)@γ-Al_2_O_3_	γ-Al_2_O_3_			93.43%	[114,115]
HPA/IL@MIL-100(Fe)	HPA			167	[101]
HPA/IL@ZIF-8	HPA			65	[101]
PTA@MIL-101(Cr)	PTA			136.5	[116,117]
PWA/MIL-101(Cr)	PWA			0.35	[65]
